# Multi-modal deformation and temperature sensing for context-sensitive machines

**DOI:** 10.1038/s41467-023-42655-y

**Published:** 2023-11-18

**Authors:** Robert Baines, Fabio Zuliani, Neil Chennoufi, Sagar Joshi, Rebecca Kramer-Bottiglio, Jamie Paik

**Affiliations:** 1 School of Engineering & Applied Science, Yale University, 9 Hillhouse Avenue, New Haven, CT 06520 USA; 2 School of Engineering, Ecole Polytechnique Fédérale de Lausanne, EPFL STI IGM RRL MED 1 2313 Station 9, Vaud, 1025 Switzerland

**Keywords:** Mechanical engineering, Polymers

## Abstract

Owing to the remarkable properties of the somatosensory system, human skin compactly perceives myriad forms of physical stimuli with high precision. Machines, conversely, are often equipped with sensory suites constituted of dozens of unique sensors, each made for detecting limited stimuli. Emerging high degree-of-freedom human-robot interfaces and soft robot applications are delimited by the lack of simple, cohesive, and information-dense sensing technologies. Stepping toward biological levels of proprioception, we present a sensing technology capable of decoding omnidirectional bending, compression, stretch, binary changes in temperature, and combinations thereof. This multi-modal deformation and temperature sensor harnesses chromaticity and intensity of light as it travels through patterned elastomer doped with functional dyes. Deformations and temperature shifts augment the light chromaticity and intensity, resulting in a one-to-one mapping between stimulus modes that are sequentially combined and the sensor output. We study the working principle of the sensor via a comprehensive opto-thermo-mechanical assay, and find that the information density provided by a single sensing element permits deciphering rich and diverse human-robot and robot-environmental interactions.

## Abstract (web summary)

Future robots require compact sensing architectures capable of discerning multiple stimuli. Here, Baines et al., present a multi-modal deformation and temperature sensor which, exploiting the light-to-state mapping, discerns combined stimuli of bending, stretching, compression, and temperature.

## Introduction

Multi-modal sensing—detecting and responding to a variety of physical stimuli—is a hallmark of human perceptive capabilities, yet has proven difficult to replicate in machines. Soft sensors have striven toward human levels of perception by serving as synthetic analogs to biological nervous system receptors^1–3^. Work on soft sensors underscores the challenge of diversifying what state variables can be artificially perceived while simultaneously reducing sensing infrastructure complexity.

The most common state variable measured by soft sensors is deformation. Numerous proposed soft deformation-sensing technologies exist; among these, a large proportion utilize conductive hyperelastic composites that change in resistance or capacitance as a function of strain^4–8^. With a single conductive sensor, it is difficult to discern multiple deformation modes. Arrangements of multiple sensing elements may also be used to expand the range of detectable modes^9–12^, but can burden systems with additional mass, fabricational complexity, and often reliance on computationally expensive data-driven models to interpret sensor outputs.

An emerging class of soft optical-based deformation sensors has several advantages over conductive sensors, including resilience to electromagnetic interference, and greater relative information density that can be gathered from light (i.e., intensity, spectral wavelength, polarization). Harnessing light intensity^13^ or intensity and wavelength together^14–17^ has resulted in detection of many deformation modes from the μm to the cm scale. Heterogenous sensing mechanisms combining optics, fluidics, and conductive material packaged within a single sensor body have been deployed to discriminate several individual and combined deformation modes via a learned model^18^. However, there is still no sensor architecture capable of distinguishing omnidirectional bending, stretch, compression, and combinations thereof with a single sensing element. Omnidirectional bending, specifically, is a ubiquitous deformation mode. It occurs among natural organisms and artificial systems, the latter including soft joysticks and soft robotic manipulators^19–21^. Knowledge of bending angle and direction is critical for effective control of such systems, but typically requires many sensors^22,23^.

In order to step machines toward biological levels of adaptive behavior, next-generation sensors must decode more than just types of deformation. In human skin, mechanoreceptors can provide both temperature and deformation sensing. Temperature sensing past a threshold can trigger myriad processes, including perspiration, pain, and shivering. Similarly, in emerging soft machines, temperatures past critical thresholds can serve as an impetus for material modulus changes^24,25^, shape-morphing^26,27^, actuation^28,29^, and self-healing^30,31^. Combined soft temperature and deformation sensors have been proposed but frequently rely on materials that are toxic or fail under cyclic loading, restricting their application space^25–35^. Optical sensing of temperature and very small uni-axial stretches in tandem (<0.1%) has been demonstrated in conventional optic fibers by exploiting Brillouin loss^36,37^; other technologies for combined temperature and deformation sensing similarly are usually viable under uni-axial strains^38,39^.

To expand the perceptive abilities of machines, we created *ChromoSense*: a sensing architecture capable of distinguishing omnidirectional bending, compression, stretch, discrete changes in temperature, and valid sequentially applied combinations thereof. The sensor comprises patterned elastomer doped with colorful dyes, including a thermochromic species (changes color when heated past a transition temperature). Deforming the sensor alters the path length of light through the colorful sections; frustrated total internal reflection results in a light chromaticity and intensity measured by an embedded miniature spectral meter. Temperature is detected as a function of the shift in spectral transmittance of the thermochromic species. Encoding dense yet disparate sensing modalities within a single soft sensor via a cohesive medium, ChromoSense provides a foundational advance toward sensing for human–machine interfaces and robots.

## Results

### Sensor design

**Fig. 1:**
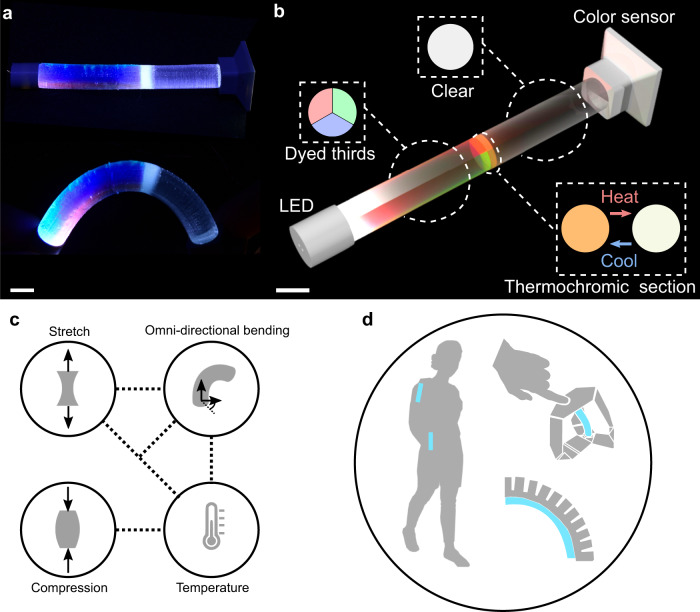
ChromoSense: multi-modal deformation and temperature sensing. **a** ChromoSense operates by measuring light wavelength and intensity as it travels through sections doped with functional dyes. Scale bar: 1 cm. **b** Generalized components of the ChromoSense architecture: an LED shines into a composite beam, consisting of dyed thirds, a thermochromic section that change chromaticity as a function of temperature, a clear section that additively mixes the light, and a miniature color sensor. Scale bar: 1 cm. **c** The proposed architecture can discern stretch, compression, omnidirectional bending, temperature, and combinations thereof indicated by the connectivity of the dotted lines. **d** ChromoSense enables myriad applications spanning wearables, human-robot interfaces, and soft stimuli-responsive machines.

ChromoSense is a stretchable cylinder made from dyed sections of optically transparent rubber, connected between rigid 3D-printed interfaces (Fig. 1 and Supplementary Fig. 1). The top rigid interface holds a white LED with a 120° transmission angle. The bottom rigid interface houses a miniaturized spectral sensor (see Note M1 for more fabrication information). Light from the LED travels inside the beam, entering three sections that experience total frustrated internal reflection (TFIR):

1. Longitudinally parallel cylinder thirds doped with red, blue, and green pigments, respectively, and bonded together. Changing the length of one of these sections relative to others, i.e., through bending, shifts the chromaticity output. Pure extension or compression, respectively, decreases or increases the light intensity output with negligible impact on chromaticity.
2. A section doped with thermochromic microcapsules in series with the polychromatic section. Occupying the entire cross-section, it does not contribute to a chromatic shift during deformations, but does only when the temperature exceeds the activation threshold of the thermochromism (Supplementary Fig. 2).
3. A neat section after all doped sections that additively mixes the light into a homogeneous output.

TFIR occurs because of the higher refractive index of the sensor material compared to its surroundings. A sensor may be clad in lower refractive index material to increase robustness in applications involving extensive environmental disturbances, otherwise no cladding is needed since air has a lower refractive index than the core material (see Note M2).

### Single-mode stimuli

**Fig. 2:**
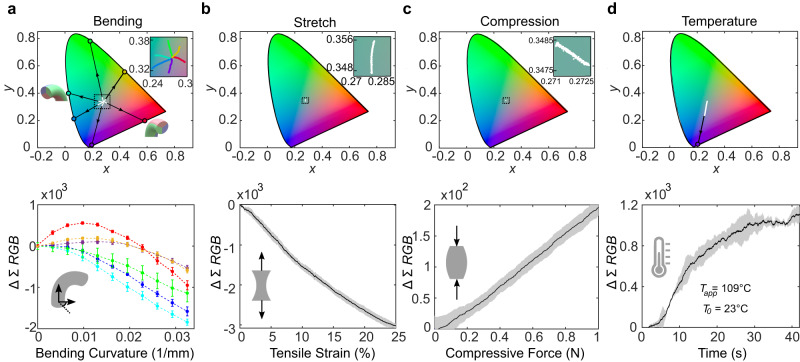
Single input mode characterization. For each subfigure, the top row shows CIE 1931 plots tracking light chromaticity, and the bottom row shows intensity. White points are experimental data and black arrows project applicable experimental data linearly to plot boundary, showing which regions of spectral saturation the bend directions tend toward. **a** Bending, where the inset zooms in to the portion of the CIE plot indicated by the black square; the color codes for each trajectory correspond to those in the intensity plot below. **b** Stretch, where negligible change in chromaticity is observed. **c** Compression, where negligible change in chromaticity is observed. **d** Temperature shift, starting from room temperature and heated with 109 °C air via forced convection until the thermochromic dye reaches a steady state (when the entire volume of thermochromic doped material exceeds the transition temperature of 31 °C). Confidence bars/clouds indicate one standard deviation for five tests with the same sample.

We subjected ChromoSense to bending in three principal directions (see Note M3 and Supplementary Fig. 3), defined as midpoints of each colorful cylindrical third, as well as bisections of those directions, while monitoring both the chromaticity visualized in the CIE 1931 color space^40^ (note that *x* and *y* coordinates are not position, but are traditional notation for chromaticity coordinates, functions of the tristimulus values^41^) and intensity of the light. We define the intensity of light as ∑*RGB*, the sum of individual *R*, *G*, and *B* components detected by the miniature spectral meter in the sensor. Results show distinct color changes corresponding to bending in principal directions and their bisections (Fig. 2a, top row). Namely, there is a chromaticity shift primarily toward the color(s) opposite the direction of bending. The black arrows projecting experimental data to the plot boundary visualize the spectral saturation toward which chromatic shift occurs. Bending that bisects principal directions results in an approximate average of the two color contributions opposite the direction of bending (see Supplementary Movie 1). Two factors contribute to bending-induced chromaticity shifts. First, light intensity increases along the side of the sensor opposite the direction of bending^42^; the colors on that side consequently contribute more to chromaticity output. Second, bending alters the path lengths of the individual colorful sections in the sensor, which act as selective wavelength filters according to Beer’s law (see Note M4 and Supplementary Fig. 4 for information about the operational principle of bending).

The additive mixing of different colors of light caused by varying the bending direction provides continuous resolution. Ellipses of increasing radius centered about the undeformed chromaticity value demonstrate this phenomenon (see Supplementary Fig. 5). Consequently, both the direction and curvature of bending may be determined from chromaticity alone. Intensity measurements when bending further reveal the mechanism of sensor operation (Fig. 2a, bottom row). The intensity output of the sensor in bending proves to be fairly repeatable, boasting less than 10% spread from the average trajectory over the entire range of curvatures and bending directions. Bending toward the maximum tested curvature generally results in a decrease in intensity because light exceeds the critical angle required for TFIR (see Notes M4 and M5).

Tension and compression (Fig. 2b, c, respectively) negligibly alter chromaticity because the relative path length through all dyed sections stays the same. There is a decrease or increase in the intensity during tension or compression, with sensitivities of 13.4 dB *ϵ*^−1^ and 2.3 dB N^−1^, respectively, where *ϵ* is strain. There is only a 2% spread in tension intensity output from the average trajectory, whereas this value is a greater 14% for compression. We attribute the lower repeatability of the sensor in compression to the different stable geometric configurations it assumes under loading.

Together, intensity and chromaticity uniquely differentiate axial deformations from pure bending modes. Cyclic stretch-and-return tests verified ChromoSense to be highly stable over 500 cycles, as it exhibited no drift in chromaticity (see Supplementary Fig. 6). Pull-to-failure experiments revealed negligible change in chromaticity until failure at a tensile strain of 61% (see Note M10 and Supplementary Fig. 7).

Heating the sensor from 23 °C past the thermochromic transition point of 31 °C alters the absorbance of the thermochromic species, desaturating it from a rich yellow (Fig. 2d). Although the thermochromism in the sensor has a binary temperature response (it does not further shift in color at temperatures higher than just above its transition temperature), the relative heat flux can be inferred through the derivative of the color shift over time as the thermochromism approaches its steady-state heated absorbance (Note M6 and Supplementary Fig. 8). The distance of CIE value shift at steady-state heated absorbance is large enough to decouple sensor readings of compound modal inputs involving temperature and bending, as will be discussed in the next section.

### Multi-mode stimuli

**Fig. 3:**
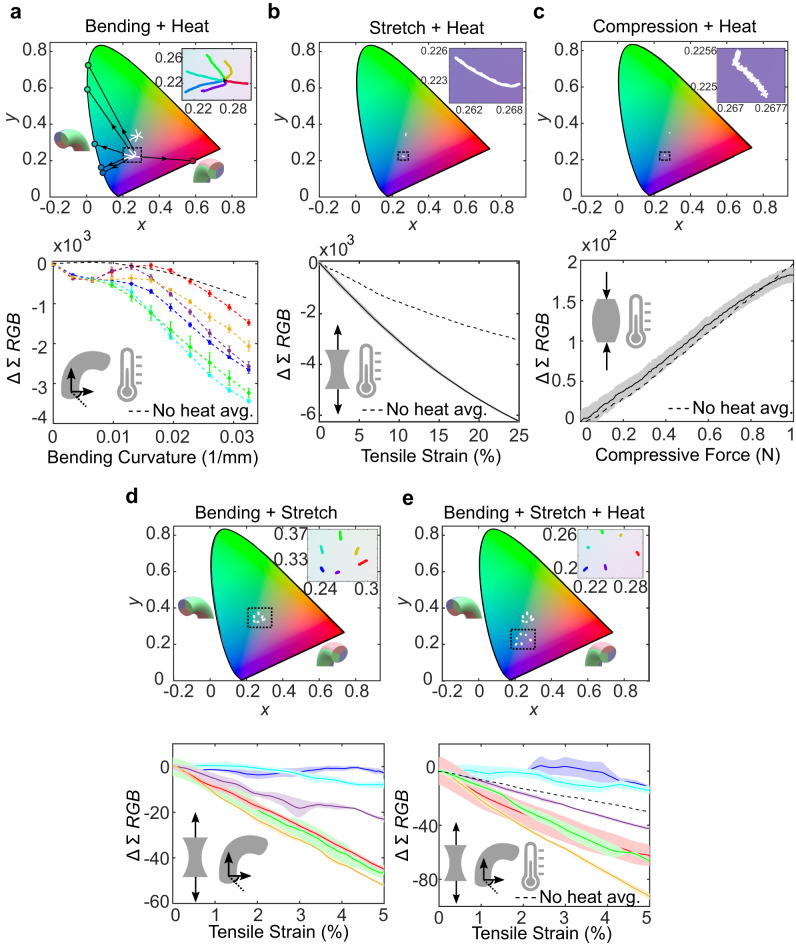
Compound input mode characterization. For each subfigure, the top row shows CIE 1931 plots tracking light chromaticity, and the bottom row shows intensity. White points are experimental data and black arrows project experimental data linearly to plot boundary, showing which regions of spectral saturation the bend directions tend toward. Tests with heat were heated with 109 °C air via forced convection until they reached steady-state heated absorbance. Then, the deformation mode was applied. Tests with heat are shifted from the analogous unheated tests, which are included in the plots for comparison. No chromaticity trajectory overlap occurs between a given heated and unheated deformation mode. Chromaticity and intensity data, together, distinguish all multi-modal cases from one another. Note that only data during the last mode in the application sequence is shown. **a** Bending + Heat, with inset showing a zoomed-in of the portion of the CIE plot indicated by the black square. **b** Stretch + Heat, with inset illustrating negligible change in chromaticity. **c** Compression + Heat, with inset again showing negligible change in chromaticity. **d** Bending + Stretch. **e** Bending + Stretch + Heat. Confidence clouds/bars indicate one standard deviation from the mean for five tests with the same sample.

To evaluate ChromoSense’s ability to discern multi-modal stimuli, we performed combined loading with bending + heat, stretch + heat, compression + heat, bending + stretch, and bending + stretch + heat. All stimuli were applied sequentially. For example, for bending + stretch, the sensor was first bent, then subjected to stretch. In cases with the addition of heat, the sensor was first brought to steady-state heated absorbance, then deformations were applied. Results attest to ChromoSense’s ability to distinguish two and even three sequentially combined modes (Fig. 3; the figure shows data during the last applied mode in a sequence).

Owing to the strong spectral shift in the thermochromic species, the chromaticity output for any mode(s) combined with heating has distinct trajectories on the CIE diagram that do not overlap with unheated ones. For example, bending + heat trajectories have no overlap with those of bending only (Fig. 3a). Likewise, heat + stretch and heat + compression are shifted in origin from the corresponding unheated modes (Fig. 3b, c). The angle splay of trajectories corresponding to bending directions on the CIE diagram resembles those in the unheated state, with the exception of the bisection of blue and red, which is skewed ~20° from the unheated trajectory, and the bisection of red and green, which exhibits a nonlinearity. We attribute these discrepancies to the change in the refractive index of the thermochromic species upon heating, which augments the critical angle required for TFIR depending on the bending direction.

Combined bending at the maximum tested curvature of 0.033 mm^−1^ with continuous strain up to 5% closely resembles chromaticity values observed in pure bending of Fig. 2a, but exhibits further decreases in intensity due to the addition of stretch (Fig. 3d). Bending + stretch + heat likewise elicits similar chromaticity to bending + heat, but with further decreases in intensity due to stretch (Fig. 3e). Importantly, since bending induces notable chromaticity and intensity changes at the same time, while stretching only induces notable intensity changes, it is possible to decouple what portion of the sensor output arises from which sequentially applied mode. Observation of the intensity value—different from the change in value shown in Fig. 3—in tandem with chromaticity facilitates decoupling (see Supplementary Fig. 9).

Application of heat generally increases ChromoSense’s sensitivity, and has negligible impact on repeatability. For example, whereas pure stretch exhibited an intensity sensitivity of 13.4 dB *ϵ*^−1^, stretch and heat yielded an elevated 15.2 dB *ϵ*^−1^. Higher relative sensitivity occurs when heated because the absorbance decreases when the thermochromism desaturates (Fig. 2d). The one exception is compression, which demonstrates comparable intensity change with the addition of heat due to the small deformation range.

Overall, for a given deformation, the addition of temperature may be decoupled from the sensor’s output because no chromaticity trajectory overlap occurs between its heated and unheated modes. A combination of intensity and chromaticity signals permits sequentially decoupling bending + stretch as well as differentiating all of the multi-modal cases from one another.

### Applications

**Fig. 4:**
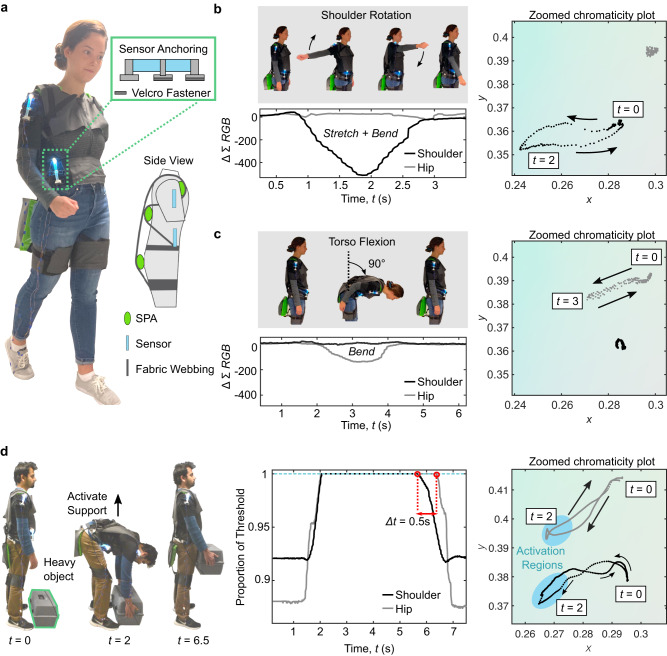
ChromoSense-enabled state estimation for a soft exosuit. **a** Torso-assisting exosuit, consisting of pouch-type SPAs placed flat on the body, overlapped by fabric straps that help to hold the SPAs against the body and transmit the forces to different regions around the torso. ChromoSenses are anchored to exosuit on the shoulder and hip with Velcro, as to enable easy repositioning to other parts of the exosuit. **b** ChromoSense detects the multi-modal 3D deformation of combined stretch and bending that occurs when the shoulder rotates, and gives in a unique CIE space value for each position in the rotation. **c** Similarly, another ChromoSense detects flexion of the torso. **d** Creating threshold regions in CIE space, we provide assistance to a user donning the exosuit when lifting a heavy object. Actuators inflate for supporting force when both sensors indicate body parts are in a lifting position with arms extended upward and outward, and torso bent forward.

Owing to its ability to robustly decipher multiple combined stimuli, ChromoSense unlocks a variety of applications in wearables, human–machine interfaces, and soft robotics. First, when placed on a soft upper body-assisting exosuit, a single sensor gives dense state feedback that can be used to infer the 3D pose of the wearer (Fig. 4a). Existing applications with soft exosuits rely on myriad sensors to estimate 3D pose, bogging down the suit with hardware and increasing computational effort to interpret sensor readings^43,44^. ChromoSense matches the compliance of the soft exosuit, and is capable of capturing the high DoF associated with natural human movement, specifically the combined continuous rotary and out-of-plane DoF at joints. For instance, whereas elbow or knee joints only require uni-axial sensing to infer pose, other joints like the hip and shoulder exhibit a broader range of motion that drives the need for multi-DoF sensing solutions.

Two sensors—one on the hip and one on the shoulder, where often complex coupled motions are observed—were used to track a wearer’s motions. Under in-plane torso flexion, the hip sensor undergoes a linear change in chromaticity. The chromaticity and intensity of the shoulder sensor remain relatively unchanged (Fig. 4b). Shoulder rotation results in a quasi-elliptical trajectory—a unique chromaticity for each shoulder configuration during rotation. The intensity signal drops significantly compared to that when bending at the torso, despite a similar displacement in CIE space of within 5%, indicating the compound stretching/bending of the sensor when the shoulder rotates (Fig. 4c and Note M7).

These baseline tests allowed us to develop threshold regions in CIE space corresponding to when the torso was bent over and the arms were extended in a grasping configuration. Color space threshold control was then used to trigger assistance lifting a heavy object (Fig. 4d, Supplementary Movie 2, and Note M7). Valuable insights about 3D pose that would require many uni-axial strain sensors typically used on soft exosuits can be gleaned from the results. For instance, plotting the proportion of threshold (capped at 1) reached as a function of time reveals a phase offset of 0.5 s between torso and arm configuration retractions when lifting the object, indicating the user’s preferential 3D body poses when lifting. In addition, the hip sensors’ CIE space trajectory exhibits a nonlinearity, suggesting the subject deviated from bending purely in-plane when picking up the object. According to the shoulder sensors’ CIE space trajectory, upon grasping the object at *t* = 2, the subject retracted and extended his arms along a different paths, though passed through a nearly identical configuration in the middle of the movement (seen as the intersection point in CIE space). Overall, the sensor provides dynamic and simultaneous biomechanical pose estimation with reduced hardware, underscoring its utility as a wearable device.

**Fig. 5:**
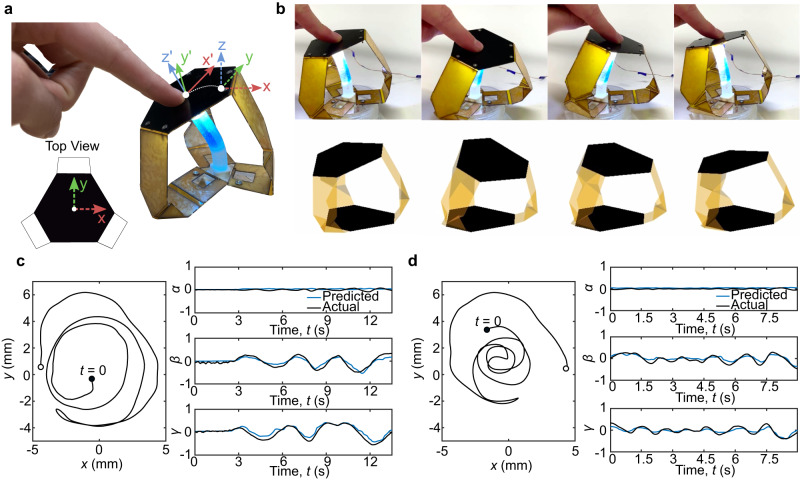
3-DoF origami interface end-effector pose estimation. **a** As the 3-DoF compliant origami user interface deforms, the *xyz* triad ascribed to its end-effector shifts to *x'y'z*'. **b** The digital twin updates real-time with the estimated configuration of the interface. **c**, **d** Left: Projected reference *x–y* coordinate system view of the interface end-effector center as the user performs a spiral motion. Right: Predicted versus actual Euler angles of the end-effector during the trajectory.

Second, ChromoSense was used to give state estimates for real-time omnidirectional bending of a compliant 3-DoF interface (Supplementary Movie 3). In the case of haptic interfaces, it is often desired to reduce complexity and footprint of sensory hardware to preserve device bandwidth and geometry. Toward this end, we placed ChromoSense within a compliant origami-inspired user interface, reducing the number of sensors required to estimate its surface orientation from at least three (on each leg) to one (Fig. 5a)^45^.

ChromoSense facilitated highly accurate, real-time simulation of a digital twin via a minimal neural network mapping chromaticity values to surface orientations (Fig. 5b and Note M8). Predicted and actual Euler angles were recorded when a user moved the interface in “out-and-back” spiral trajectories of varying speeds and deformation amplitudes. The first case of higher amplitude, lower frequency spirals shows strong agreement between Euler angles, with the root-mean-squared error of 0.0516 (radians) for *α* (roll), 0.0966 for *β* (pitch), and 0.1052 for *γ* (yaw) (Fig. 5c). The second case of lower amplitude yet higher frequency oscillations also has an excellent agreement, with the root-mean-squared error of 0.0499 (radians) for *α*, 0.0784 for *β*, and 0.0915 for *γ* (Fig. 5d). Due to the linear response of ChromoSense with deformation, and its ability to accurately capture user input across different oscillation frequencies and curvature magnitudes, it furnishes a simple and reliable means for user-in-the-loop tracking of high-DoF mechanism kinematics.

Third, we employed ChromoSense in a variable stiffness soft robotic manipulator to respond to environmental temperature changes through move-and-hold operations to two prescribed opposite-handed curvatures, denoted ± *κ*_*t*_ (Supplementary Movie 4 and Note M9). No system to date has demonstrated combined multi-modal deformation and temperature sensing for closed-loop control of a soft robotic manipulator with a single sensing element. In fact, the majority of thermally activated soft robotic systems are open-loop^46–49^.

**Fig. 6:**
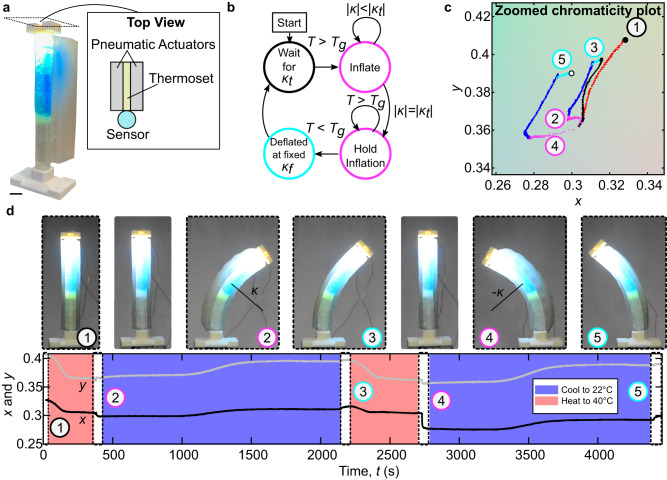
Autonomous variable stiffness manipulator. **a** Image of the variable stiffness manipulator with a top view schematic depicting the various functional layers. Scale bar: 13 mm. **b** State diagram for the program controlling the autonomous variable stiffness manipulator. **c** Chromaticity diagram of sensor integrated within manipulator during the autonomous move-and-hold sequence. Important events are enumerated on the diagram. **d** Top: pictures of enumerated events from (**c**). Bottom: Corresponding *x* (black) and *y* (gray) chromaticity coordinates plotted as a function of time, in the context of forced environmental heating or cooling, indicated by red and blue sections, respectively.

The manipulator consists of an antagonistic pneumatic actuator pair adhered together by a thermosetting polymer (Fig. 6a). The thermoset has a glass transition temperature region, *T*_*g*_, spanning 35–40 °C, which roughly coincides with the color shift temperature of the thermochromic dye in the sensor. When the manipulator is exposed to temperatures at or exceeding *T*_*g*_, the modulus of the thermoset drops 4 × , allowing deformation by the actuators. The sensor was bonded along the outside of the thermoset-actuator assembly to facilitate temperature and bending curvature sensing and to decouple it from pressure-induced surface strains.

We devised an autonomous control policy for the manipulator (state machine depicted in Fig. 6b). This move-and-hold sequence was seeded with ± *κ*_*t*_, empirically specified a priori via chromaticity values at deformed states of interest. Temperature changes were induced via forced air in an environmental chamber.

Chromatic trajectories during the move-and-hold sequence underscore ChromoSense’s ability to effectively interpret temperature and bending state variables simultaneously. Individually plotted chromaticity coordinates (*x* and *y*) as a function of time hone in on more detail of when each step of the move-and-hold sequence occurred (Fig. 6c, d; note that subfigures c and d highlight key states via contour color-coding that match those of subfigure b). The move-and-hold sequence starts with the manipulator at 22 °C (Fig. 6d1). Environmental heating to 40 °C then induces a strong chromaticity shift in the thermochromic species, indicated by the red line. ChromoSense registers when the temperature is above *T*_*g*_, triggering inflation of an actuator (Fig. 6d2). Consequently, chromaticity shifts leftward on the green line as the actuator strives to attain *κ*_*t*_. Upon reaching *κ*_*t*_, pressure is held constant. As the environment cools, shown by the blue line, ChromoSense registers when the temperature drops below *T*_*g*_, triggering deflation of the actuator. Curvature is maintained in the absence of pressure; however, the restoring spring force of the manipulator reduces its magnitude by 20%, and the corresponding chromaticity shift is evidenced by the cyan line (Fig. 6d3). This concludes half of the move-and-hold sequence. Heating the environment beyond *T*_*g*_ again causes the manipulator to relax back to its undeformed, heated configuration along the black line. The ensuing triggered inflation along the green line (Fig. 6d4), cooling along the blue line, and release along the cyan line (Fig. 6d5) concludes the sequence for the two specified curvatures of equal magnitude and opposite sign.

## Discussion

ChromoSense harnesses parallel and series patterns of elastomer doped with functional dyes to detect rich contact and non-contact stimuli—all in a single sensor. These stimuli include stretch, compression, omnidirectional bending, discrete changes in temperature, and valid sequentially applied combinations thereof, that may be distinguished via chromaticity and intensity changes. One ChromoSense can replace arrays of conventional sensors for state feedback of wearables, human interfaces, soft robots, and prospectively a vast array of other high-DoF mechanisms. As an illustration of such versatility and utility, we presented three demonstrations.

First, a soft exosuit was outfitted with two ChromoSenses to estimate 3D pose, providing insight into wearer biomechanics. ChromoSense matches not only the DoF of the exosuit, but its compliance as well. We then utilized a novel color space threshold control to trigger force-assisted object lifting. Results indicate ChromoSense has significant ramifications for low-footprint wearable technologies.

Second, an origami interface was equipped with one ChromoSense to estimate its orientation in 3D space, accurately updating a digital twin in real time. We substantially reduce the hardware required for state information updates, enabling seamless user interface experiences. Moreover, this demonstration showcased how state-of-the-art predictive models like neural networks can be applied to ChromoSense’s output to obtain high-resolution estimates of bending magnitude and direction.

Third, one ChromoSense was used to facilitate autonomous, simultaneous thermal signal processing and curvature control of a variable-stiffness robotic continuum manipulator that would traditionally require deformation sensors and separate temperature sensors to achieve. The ability to decouple multiple modes of stimuli in tandem elevated the information density, enabling the manipulator to respond adaptively to the environment.

Leveraging intrinsic optical properties of sensor host materials allows us to not only capture rich and diverse stimuli, but also avoid explicitly accounting for complicated properties like strain rate-dependence, electromigration, and Mullins’ effect that can be issues in conductive composite sensors. The ChromoSense design paradigm is therefore well-disposed to multiple scales and amenable to numerous designs beyond those presented herein (see Note M2 for a discussion about trade-offs of certain design variables). Although ChromoSense has a limitation in terms of its ability to decouple simultaneously applied stimuli, we suspect that there are several data processing approaches that could be used to address this in future work (see Note M10). As one extension to the present work, we foresee that temperature could possibly be detected as a continuous variable if using a chemical species that exhibits a continuous spectral shift as a function of temperature, like thermochromic liquid crystals. Overall, by packing multi-modal sensing functionality into a single robust sensing element, we take the next leap toward biological levels of proprioception.

## Methods

### Note M1: ChromoSense fabrication details

An exploded view detailing one specific sensor embodiment’s components is presented in Supplementary Fig. 1. Sensors were fabricated first by pouring polymer into 3D-printed (Formlabs, Form 3+) molds to cast the cylindrical thirds, each being 50 mm long. We made two types of sensors. The first used Polydimethylsiloxane (PDMS) mixed in a 10:1 ratio by mass, and the second employed urethane (smoothON Inc., ClearFlex 30) mixed in a 10:9.4 ratio by mass. The specific concentration of dyes was: R - 1drop/11g, B - 3 drops/11g, G - 7drops/11g (EPODEX, Transparent Red, Blue, and Green—Drop-In Farbstoff). In both cases, polymer constituents were mixed together for 2 min, de-gassed for 3 min, and left to cure at 23 °C for 24 h. Next, we bonded the thirds together by using a thin layer of the same polymer. Then, the finished tri-color cylinder was placed into another mold and polymer doped with the thermochromic microcapsules (Adafruit, Thermochromic Pigment–Yellow–10 g) was poured in to create a layer of 1 mm height section in series. Finally, an additional 50-mm-thick layer of polymer without any dye in it was deposited on top of the thermochromic section. The resulting sensor is 101 mm long and 10 mm in diameter. The LED (OSLONSignal, LUW CRBP.01) and color sensor (Adafruit, TCS34725) are constrained by parts 3D-printed from Acrylonitrile butadiene styrene (ABS). The color sensor mounted on a signal processing board was press-fit into the color sensor holder. The particular dimensions of these sensors were chosen based on length scale and the applications, though many different geometries are possible. We discuss scaling considerations in Notes M2 and M4.

### Note M2: ChromoSense design considerations

The specific ChromoSense embodiment of Supplementary Fig. 1 and subsequently employed for several demonstrations had design parameters that were empirically determined. These parameters are not necessarily optimal for the chosen geometry, so a more rigorous parametric study must be conducted to identify optimal parameters. In our case, basic physical principles combined with a quantitative assay of sensor performance guided this parameter selection. Key performance metrics included (1) intensity of light at the output, quantified via measuring a proxy, the sum of RGB components from the color meter; (2) distinctness of chromatic responses to a given mode, quantified by measuring the dissimilarity between modal responses (i.e., for bending, the angle between CIE swept paths in principal directions); (3) sensitivity, quantified by the magnitude of RGB ratio changes at maximum bending angle; (4) stiffness and stretch at yield, determined through uni-axial quasi-static tension tests.

The theoretical maximum length of the entire ChromoSense depends firstly on the luminous flux output from a given LED. Longer sensors will generally require LEDs omitting higher luminous flux in order to transmit light down the entire length, since light diminishes in power quadratically per unit distance. Shorter sensors will therefore have lesser flux requirements. Optical properties of the sensor material will further dictate the required power of an LED. Materials that exhibit high absorbance, scattering, and reflectance will mandate higher power. Similarly, the concentration of functional dyes within the material must be tuned to allow enough light through, while simultaneously serving as selective wavelength filters.

If any modes are to be associated with a unique sensor output chromaticity in the chosen CIE embedding, (1) deformations must lead to unique path lengths through colorful sections, and (2) the spectral shift exhibited by the thermochromic section must prevent deformations in heated and un-headed conditions from overlapping. The former is addressed via the geometric configuration of the different ChromoSense sections as parallel and series assemblies, as discussed in the main text. The latter entails selecting an appropriate concentration and volumetric occupancy of the thermochromic species to allow sufficient absorbance, as well as choosing a thermochromism with spectrally distinct heated and unheated phases. There are numerous commercially available thermochromic substances, spanning organic dyes to liquid crystals. They are available for a wide range of operational temperatures from −100 to 200 °C^50^. Organic dyes often come in the form of easily dispersible microcapsules, such as the ones used in this paper, and exhibit a binary color change above a temperature threshold. Mixes of thermochromic dyes can exhibit several color changes at different threshold temperatures. Liquid crystals exhibit essentially continuous change in color as a function of temperature; a typical application of these is to assess heating distribution of electronic components. With such an abundance of thermochromic compounds, creating ChromoSense units capable of higher resolution temperature and deformation sensing is certainly feasible.

Lastly, as with conventional optical fibers, the chosen material for the ChromoSense core must have a higher refractive index than its surroundings, or cladding, in order to produce total frustrated internal reflection. Two different polymeric, stretchable materials—Sylgard 184 (DOW Chemical) with a refractive index of 1.41, and ClearFlex 30 (SmoothOn Inc.) with a refractive index of 1.46—were used to make sensors during explorations for the present paper. The refractive index of air is 1. In applications where a sensor does not experience significant contact with other surfaces, it suffices to proceed without additional material cladding. For related optical sensing architectures, we refer the interested reader to a collection of pertinent materials, their refractive indices, and Young’s moduli as in reference^17^.

Overall, a number of coupled physical parameters and their combination dictate the performance of a ChromoSense: dimensions of the sensor sections, optical and mechanical qualities of the materials, concentrations of functional dyes, and thermochromic capabilities of dyes. A wide design space exists, in terms of different sensor architectures, that could even expand sensed deformation modes.

### Note M3: multi-DoF characterization setup and other characterization tests

We used a platform consisting of five degrees-of-freedom (DoF) to characterize the chromaticity and intensity output of the ChromoSense in a variety of pure bending modes of different curvature and direction of curvature (Supplementary Fig. 3). The platform consists of two separate kinematic mechanisms whose end-effectors are connected through the ChromoSense. The ChromoSense is mounted on the bottom to an X-Y gantry (FUYU, FSL40) and to the top to a Z-roll-pitch system (FUYU, FSL40 connected to two Dynamixel, AX-12).

To prescribe sensor centerline curvature *κ*, length *L*, and direction of curvature *ϕ* for characterization, we developed representations for each kinematic mechanism via homogeneous transformation matrices arising from the modified DH-parameters^51^ below:

**Table:** 

Link (PP)	*α*_*i*−1_	*a*_*i*−1_	*d*_*i*_	*θ*_*i*_
1	0	0	280-*d*_*x*_	0
2	*π*/2	85	120-*d*_*y*_	*π*/2
3	*π*/2	0	50	0

**Table:** 

Link (PRR)	*α*_*i*−1_	*a*_*i*−1_	*d*_*i*_	*θ*_*i*_
1	0	0	$275-{d}_{z}$	0
2	$-\pi {{{{\mathrm{/2}}}}}$	0	120	${\theta }_{2}-\pi {{{{\mathrm{/2}}}}}$
3	$-\pi {{{{\mathrm{/2}}}}}$	0	0	${\theta }_{3}-\pi {{{{\mathrm{/2}}}}}$
4	0	50	0	0
5	$-\pi {{{{\mathrm{/2}}}}}$	0	−40	0

Here, [*d*_*x*_, *d*_*y*_, *d*_*z*_] and [*θ*_2_, *θ*_3_] are the controlled kinematic parameters. The sensor was modeled as a centerline curve assumed to be of constant curvature, determined by the displacement between the two end-effectors. Kinematic parameters to achieve desired κ^*^ and ϕ^*^ were computed by minimizing:

1 $$\mathop{\min }\limits_{{d}_{x},{d}_{y},{d}_{z},{\theta }_{2},{\theta }_{3}}\,{(\kappa -{\kappa }^{*})}^{2}+{(\phi -{\phi }^{*})}^{2}+{{{{{{{\mathcal{D}}}}}}}}(\overline{T}\times \overline{PRR})+{{{{{{{\mathcal{C}}}}}}}}{(L-{L}^{*})}^{2}$$

2 $$s.t:Lb\le {d}_{x},\, {d}_{y},\, {d}_{z},\, {\theta }_{2},\, {\theta }_{3}\le Ub$$

Here, $\overline{T}$ is the tangent vector at the top of the sensor and $\overline{M}$ is the tangent vector of the grips (cross product tending to zero enforces alignment with the grips), the * superscript denotes user-input goal values, and ${{{{{{{\mathcal{C}}}}}}}}$=100 and ${{{{{{{\mathcal{D}}}}}}}}=$1000. Note all variables in the objective are functions of the kinematic parameters. Boundary conditions and length are conditionally weighted to enforce solutions for pure bending without additional stretch or compression of the centerline. Minimization was performed with a genetic algorithm coded in MATLAB. The resulting outputs were converted to the motor commands and implemented on the setup to achieve programmed deformations.

We conducted tension and compression experiments on the sensor using an Instron 3345 fitted with a 1 kN load cell. All tests were conducted at a loading rate of 40 mm/min. For sensor characterization tests depicted in Figs. 2 and 3, we applied deformations considering a factor of safety on failure, so we could test the same specimen multiple times and quantify repeatability in the operational range of the sensor. Compression was applied up to just before the buckling force of the sensor. In uni-axial experiments, tension was applied up to 25% strain (the pull-to-failure results are discussed in Note M10). For combined bending and stretch tests, we used the Instron machine outfitted with a custom fixture to enforce a fixed, maximum curvature while stretching (Supplementary Fig. 9a). We set the maximum axial strain to 5%. Note that certain physically unrealizable combined modes of deformation were not tested. These unrealizable combined modes include compression + bending, because the sensor cannot maintain a fixed curvature with additional compression boundary conditions, as well as compression + stretch, since both conditions act along the axis of the sensor and effectively cancel each other out. For tests involving heat, we applied heat at 109 °C via forced convection until the chromaticity value reached a steady state. Steady state occurs when the entire volume of doped material exceeds the transition temperature (31 °C) of the thermochromic species.

### Note M4: working principle of ChromoSense in bending

Two factors help explain chromaticity shifts that occur during bending: (1) ray path propagation concentration; (2) path length modulation of individual colorful sections in the sensor that act as selective wavelength filters. Regarding the first factor, it has been shown that in bent fiber optic cables, light rays concentrate at the side opposite to the bending direction. Ray concentration results in an increase in light intensity at that side and a decrease in intensity at the side toward the direction of bending^42^. In the case of a bent ChromoSense, colors opposite the direction of bending will have relatively greater light intensity coming through them, amplifying their contribution to overall chromaticity.

To explain the second factor, ChromoSense was modeled as a 2D beam in pure bending, assuming homogeneous and linear materials. The axial strain of the beam when under curvature is given as:

3 $${\epsilon }_{z}=-\kappa Rsin(\theta )$$

Here, *κ* is the radius of curvature, *R* is the distance from the neutral axis to a point in the beam cross-section, and *θ* completes the polar coordinate system. This equation elucidates that axial strain varies linearly as a function of distance from the neutral axis, reaching a minimum opposite (180° in *θ*) of the direction of bending, and a maximum in the direction of bending (0° in *θ*). For example, when bending directly toward the intersection of the blue and green thirds, red stretches maximally, and the majority of blue and green are compressed. The result of these multi-color sectional stretches on chromaticity can be understood via Beer’s law^52^, written as:

4 $$A=\epsilon c\ell$$

Here, *ϵ* is the molar (decadic) absorption coefficient, *c* is the concentration of solution, *ℓ* is the path length through a medium, and *A* is the resulting absorbance. As *ℓ* increases, so does the absorbance of the medium. Yet absorbance is not uniform across all wavelengths; indeed, stretching monochromatic (RGB) beams, we validated that the output chromaticity shifts toward the beams’ color (i.e., stretching a red beam, the ratio of red increases in the output).

Specifically, we conducted tensile tests (Instron 3345 fitted with a 1 kN load cell) on 50 mm-long and 10 mm diameter PDMS beams doped a single color, red (1 drops/11 g polymer), green (3 drops/11 g polymer), or blue (2 drops/11 g polymer). We applied a strain of 10% at a rate of 100 mm/min. Results illustrate how chromaticity change is linear with displacement, and increases toward the color of the dyed beam (Supplementary Fig. 4, top). For example, the blue beam shifts towards blue as it is stretched. The clear beam exhibits negligible change in chromaticity when stretched. Linearly fit slopes of intensity change for red, green, blue, and clear beams were −907, −791, −552, and −504 units/mm, respectively. Differences in slope values can be attributed to the different concentrations of dye used for each beam and different absorbance characteristics of the dye. Signal intensity decreases linearly and monotonically with displacement. Therefore, under this model, a color contributes maximally to the chromaticity of the light output when the section corresponding to that color is opposite the direction of bending because it acts as a wavelength-selective filter of increased *ℓ* and the colors opposite it decrease in *ℓ*. We contend that path length modulation impacting the light filtering, as described by Beer’s law, as well as ray path propagation concentration, together explain the chromatic response in bending.

Based on the combined optical physics explanation for bending, we expect that the radius of the ChromoSense should scale in proportion to its sensitivity in bending, because more dyed material is pushed away from the neutral axis and will thus stretch more under the same radius of curvature. By the same logic, decreasing the ChromoSense’s radius will be possible only to a certain limit governed by color meter’s ability to still distinguish changes in chromaticity. For the current ChromoSense embodiment, the sensitivity in bending depends on the direction of bending, inclusion of heat, and the regime of curvature. For example, when the blue section is predominately stretched, there is a sensitivity of 3.97 dB mm^−1^. If the green section is predominately stretched, this value is 2.24 dB mm^−1^. When averaged over the three principal directions and their bisections in the curvature regime of 0.02 to 0.03 mm, sensitivity comes out to 3.31 dB mm^−1^. In the case of bending + heat, this average is an elevated 5.72 dB mm^−1^. This variance in sensitivity is also accompanied by inconsistently monotonically decreasing behavior for certain bending directions. Bending such that blue lies within the stretched region of cross-section results in a monotonically decreasing intensity versus curvature. On the other hand, bending such that red lies within the stretched region of cross-section results in an initial increase then decrease in intensity. We attribute these phenomena to the facts that: (1) the concentrations and absorbance of each color dye are different (Note M5 presents UV–vis characterization); (2) the LED may not be perfectly emitting uniform radiation; and (3) the refractive index of the materials varies due to different chemical dopants, causing different wavelengths and quantities of light to escape the sensor when bending based on the bending direction.

### Note M5: UV–vis spectra of dyed polymers

Normalized spectral transmittance of doped polymer at different wavelengths of light was determined using a UV/Vis Spectrophotometer (LAMBDA, 365) on three different samples doped with red, green, and blue dyes (EPODEX, Transparent Red, Blue, and Green—Drop-In Farbstoff). Results attest to the highly selective filtering effect the dyes have on light passing through them (Supplementary Fig. 2a). The change in color of the thermochromic dye was quantified via transmittance as well; these curves show how heating past the transition threshold allows more light in general, and proportionally more light from 550 nm wavelengths and upwards to pass through (Supplementary Fig. 2b). Optically, a change from yellow to off-yellow occurs when the dye is heated; such color changes in fluoran-based thermochromic compounds are triggered by a temperature-dependent proton transfer between the acidic developer and the fluoran^53^ (Supplementary Fig. 2c).

### Note M6: thermal characterization experiments

We used a heat gun (Steinel HG 1910 E) 5 mm away from an ChromoSense and applied different temperatures to study the chromaticity shift it underwent. Higher temperature resulted in a faster change in chromaticity, as quantified by the slope of the RBG ratio components (Supplementary Fig. 8). For example, *T*_*app*_ = 169 °C resulted in faster chromaticity change than heating at *T*_*app*_ = 49 °C. The speed of color change is consistent with the mechanism governing temperature-dependent color changes in fluoran-based thermochromic compounds: the sooner the energy barrier for proton exchange is reached, the sooner the color change occurs^53^. The slope of the color components can therefore be used as an indication of heat flux, potentially expanding the utility of the sensor to dynamic temperature sensing scenarios.

### Note M7: soft exosuit

The soft exosuit consists of donned fabric vest with spanning mesh straps that transfer force from soft pneumatic actuators (SPAs) on the shoulders and back to provide postural assistance. ChromoSenses were mounted in a 3D-printed legs with Velcro fasteners on the bottoms to anchor them to the exosuit in various locations. The anchors for the shoulder joint contain three Velcro fasteners, one on each leg. Due to the location of the shoulder joint relative to the sensor placement, the sensor length increases upon rotation of the shoulder, resulting in simultaneous stretch and bending. Torso flexion causes the length of the sensor on the hip to decrease, thus the sensor buckles if all three legs are anchored. The buckling is an instability whose direction we cannot control, so we decided to have the top of the sensor move freely without axial compressive forces by removing the top Velcro fastener.

A pressure regulator (SMC ITV1011-21F1N) was triggered via PWM signal generated from an Arduino to inflate or deflate the pouch actuators depending on the state estimation provided by the ChromoSenses. The pressure line was supplied with 20 kPa. The authors affirm that human research participants provided informed consent for publication of the images in Fig. 4.

### Note M8: origami interface

The origami interface shown in this work is a parallel platform consisting of three legs. It is fabricated through layer-by-layer manufacturing and employs the combination of several functional layers. Rigid parts made of glass fiber are linked together by a flexible Kapton layer. The distribution of flexible layer “joints” allows the structure to exhibit compliance at specific, programmed locations. We laser-cut parts for each layer and heat-bonded them together with solid adhesive. For more information about the origami interface, please see ref. ^45^. The three-legged self-standing platform is retrofitted with the ChromoSense between its end effector and base, reducing the needed sensors to determine any movement from at least three (for each joint) to just one in the center of the interface.

For digital twin demonstrations, we trained a two-layer neural network using MATLAB fitnet to associate a given RGB ratio triplet from the ChromoSense to the interface’s top surface orientation. The neural network includes one hidden layer with a sigmoid activation function, and one output layer with a rectified linear unit output function. The input dimension is a 3 × 1 vector of RGB values, and the output is a 3 × 3 rotation matrix flattened to a 9 × 1 vector. The training was performed with an Aruco marker to determine the ground-truth orientation of the surface. We used the trained neural network to map RGB values to an orientation so as to update a digital twin of the interface in real time. The graphics simulation was coded with TKinter^54^ and uses online optimization to compute the inverse kinematics of the structure.

Owing to the sensor’s one-to-one mapping between any bending deformation and a chromaticity value, the model facilitated excellent matching between the digital twin and experiments. These experiments verified that a minimal data-driven model is an effective way to map sensor outputs to desired units of deformation.

### Note M9: autonomous variable stiffness manipulator

We created the variable stiffness manipulator by casting two pneumatic actuators from Dragon Skin 10 (SmoothOn Inc.), and bonding them to muslin fabric strain-limiting layers. These two actuators were then conjoined along their strain-limiting layers via a 1-mm-thick thermoset polymer. In particular, a 10:4 by mass ratio of Jeffamine D400 (Huntsman International) and Epon 828 (DOW Chemicals) was mixed thoroughly for 2 min, degassed for 10 min, and poured into a mold placed into an oven at 38 °C for 12 h. One ChromoSense was bonded to the *x–y* plane neutral axis of the manipulator with Silpoxy (SmoothOn Inc.).

The manipulator assembly was placed within an environmental chamber outfitted with heating elements, and pressurized air for cooling. A digital thermocouple within the chamber provided ground-truth temperature readouts during the experiments. We used miniaturized pneumatic pressure regulators^55^ to inflate the actuators on the manipulator.

In the state machine depicted in Fig. 6b, state transitions take place if temperature and curvature are detected to pass specified thresholds. Threshold detections are implemented as inequalities or equalities. Values used in the expressions are empirically determined CIE coordinates associated to the actual variable. For instance, the state transition requiring ∣*κ*∣ = ∣*κ*_*t*_∣ indicates that the present curvature *κ* of the sensor equals the target deformed curvature *κ*_*t*_. We determined a chromaticity coordinate [*x*, *y*] for each of *κ* and *κ*_*t*_ in a calibration phase prior to running tests. As another example, the state transition *T* > *T*_*g*_ indicates that the temperature *T* of the manipulator exceeds the glass transition *T*_*g*_ of the variable stiffness material embedded within it. *T* is estimated to be greater than *T*_*g*_ because the thermochromic portion reaches a steady-state heated absorbance, indicating the manipulator is above 31 °C. As with curvature detection, chromaticity coordinates are determined at steady-state heated absorbance and unheated states, and used in threshold expressions in the state machine.

### Note M10: limitations of ChromoSense in this paper

The remainder of the methods section will discuss limitations of the PDMS ChromoSense embodiment used for characterization in the present paper, and suggest possible avenues for addressing these limitations. Under uni-axial tension, the ChromoSense has a lower detection limit of 0.02 mm. A pull-to-failure test reveals a continuing decrease in intensity as a function of stretch, and maintenance of negligible change in chromaticity (see Supplementary Fig. 7). The stress in the sensor cross-section (calculated with respect to the reference area) exhibits non-linearly increasing behavior until failure, at around 61% strain, when there is a sudden decrease in stress and intensity as the sensor is split in two. Failure tends to occur along the adjoining colorful sections or at the interface between the thermochromic portion and the clear portion. We found that increasing the doping concentrations of the dyes embrittles the ChromoSense, advancing the onset of failure, and increasing the force response during deformation. One possible solution to improve the elongation at failure is to adjoin sensor sections—cylindrical thirds, thermochromic portion, and clear portion—using integrated manufacturing processes such as 3D printing, rather than individually casting and then bonding them. To combat the dyes’ embrittling effects, lower concentrations could be employed, but the sensor geometry must be scaled appropriately to retain the desired net filtering effect.

During bending, the lower detection limit depends on the direction, and is, on average, 3 × 10^−4^ mm^−1^. There is a saturation limit around 150° (the exact value depends on the specific direction of bending and the predominately stretched and compressed color portions) past which the expected trend in chromaticity shift stops. Namely, past the saturation limit, the color content of the output signal no longer increases toward those colors on the convex side of the sensor. We hypothesize this behavior arises because the critical angle required for TFIR has been exceeded, and light can readily escape the sensor. One possible way to improve the maximum sensed bending angle is to further increase the refractive index of the sensor relative to its surroundings through choice of other materials or claddings.

Another point of note is that the sensor’s baseline chromaticity is quite sensitive to dye concentrations. In practice, we found that slight variations in RGB and thermochromic dye concentrations ( ± 5% by weight of a single dye) can impact resting chromaticity x and y coordinates offsets as much as ± 0.02 in each coordinate. Precise balance between dyes is needed in order to elicit a baseline chromaticity that is well-centered within the CIE diagram and undergoes approximately linear trajectories in CIE space during bending deformations.

An additional challenge with ChromoSense is that the time-dependent response of the thermochromic shift makes complete decoupling of temperature and deformation modes possible only at steady-state heated absorbance. For example, if only half of the volume of thermochromic dye transitioned and then a bending deformation occurred, we could not intuitively decouple temperature from deformation modes. One possible way to speed up the sensor’s response to temperature is to reduce the volume of the thermochromic part and correspondingly increase its concentration, decreasing its thermal mass. Relatedly, the binary response of the thermochromism prevents continuous temperature detection. ChromoSense could not distinguish between any temperature just above the thermochromic transition point and one much higher, like 40 °C and 90 °C. Consequently, the current combined temperature and deformation-sensing capabilities may not be sufficient for applications that are fast-paced or that require continuous temperature estimations.

As a last point of note, decoupling and estimating the relative contribution of various stimuli to the sensor output is possible based on knowledge of intensity, chromaticity, and their empirically determined bounds. For instance, for sequentially combined bending + stretch, bending entails changes in chromaticity and intensity at the same time, whereas stretch entails negligible changes in chromaticity, enabling us to decouple the contributions to the output signal by analyzing changes in both channels throughout applied deformations. However, ChromoSense—like any sensor—requires a model to map output values to finite strains and curvatures. A variety of optical waveguide-based sensing architectures have spurred complementary approaches to estimate specific magnitudes of stimuli from a sensor’s output. These approaches include both data-driven^15,18,56,57^ and physics-based^22,58^ models. ChromoSense exhibits nonlinearities when subject to certain combined stimuli; a chosen model must therefore account for such nonlinearities in order to be applicable over the entire operational range of the sensor. One promising avenue would be to employ a minimal recurrent neural network. Since ChromoSense provides a one-to-one mapping between any applied stimulus and the output signal, the recurrent time horizon of information could yield finite strains, curvatures, and heat presence for sequentially combined modes.

### Supplementary information

Supplementary Information

Peer Review File

Description of Additional Supplementary Files Document

Supplementary Movie 1

Supplementary Movie 2

Supplementary Movie 3

Supplementary Movie 4

## Data Availability

The origami interface workspace data generated in this study are available at the public repository https://github.com/the-faboratory/ChromoSense (10.5281/zenodo.7799503). All other data needed to evaluate the conclusions in the paper are present in the paper and/or the Supplementary Information. Additional data related to this paper are available from the authors upon request.

## Code availability

Code can be found at the public repository https://github.com/the-faboratory/ChromoSense (10.5281/zenodo.7799503)^59^.
