# Publisher Correction: Multi-modal deformation and temperature sensing for context-sensitive machines

**DOI:** 10.1038/s41467-023-44212-z

**Published:** 2023-12-15

**Authors:** Robert Baines, Fabio Zuliani, Neil Chennoufi, Sagar Joshi, Rebecca Kramer-Bottiglio, Jamie Paik

**Affiliations:** 1 School of Engineering & Applied Science, Yale University, 9 Hillhouse Avenue, New Haven, CT 06520 USA; 2 School of Engineering, Ecole Polytechnique Fédérale de Lausanne, EPFL STI IGM RRL MED 1 2313 Station 9, Vaud, 1025 Switzerland

**Keywords:** Mechanical engineering, Polymers

Correction to: *Nature Communications* 10.1038/s41467-023-42655-y, published online 18 November 2023

The original version of this Article contained an error in Fig. 3, in which the insets of Fig. 3a and 3b were not correctly visualized. The correct version of Fig. 3 is:
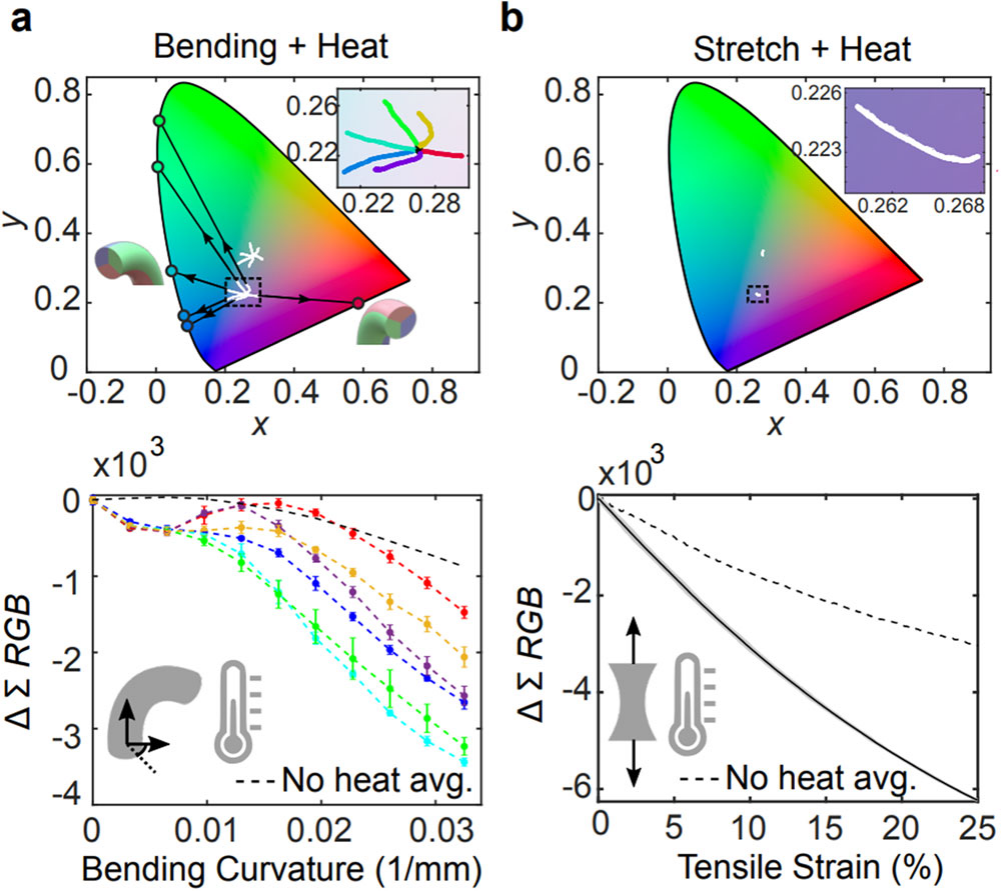


which replaces the previous incorrect version:
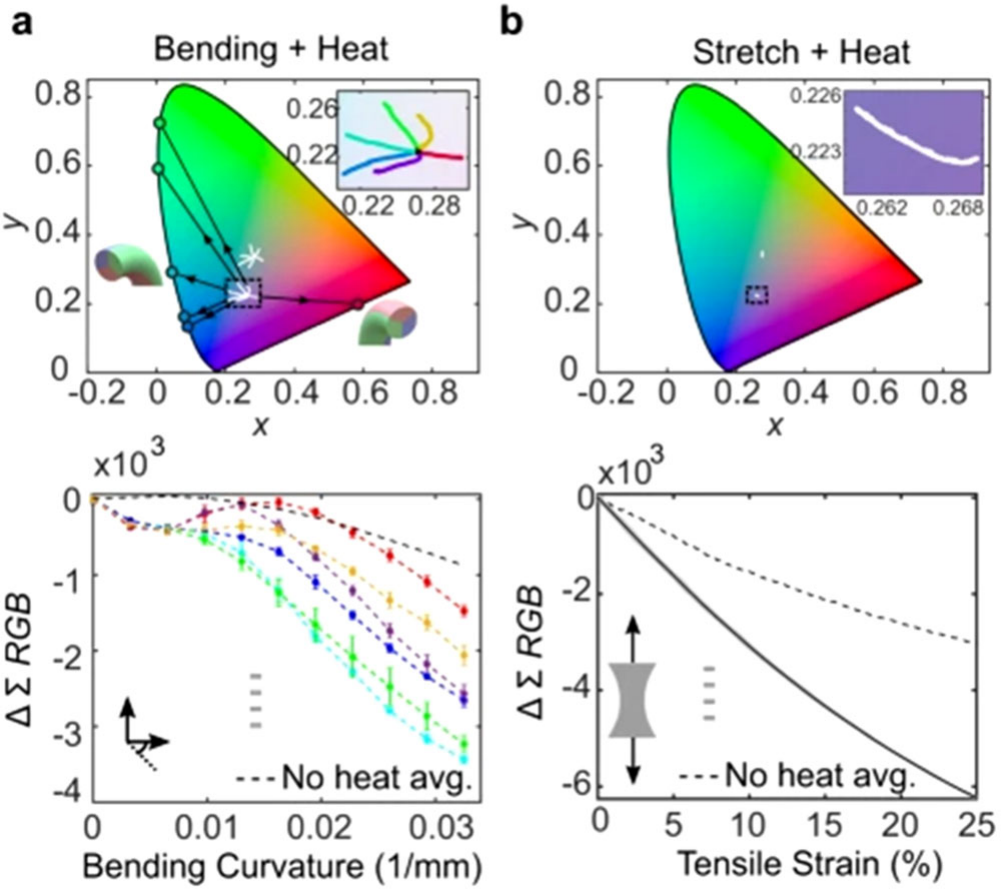


This has been corrected in both the PDF and HTML versions of the Article.

