# Supplementary material for: Multi-modal deformation and temperature sensing for context-sensitive machines
Source: Nat Commun. 2023 Nov 18;14:7499. doi: 10.1038/s41467-023-42655-y (PMC10657382; doi:10.1038/s41467-023-42655-y)
Supplement: Supplementary file 1 — Supplementary Information [file 41467_2023_42655_MOESM1_ESM.pdf]

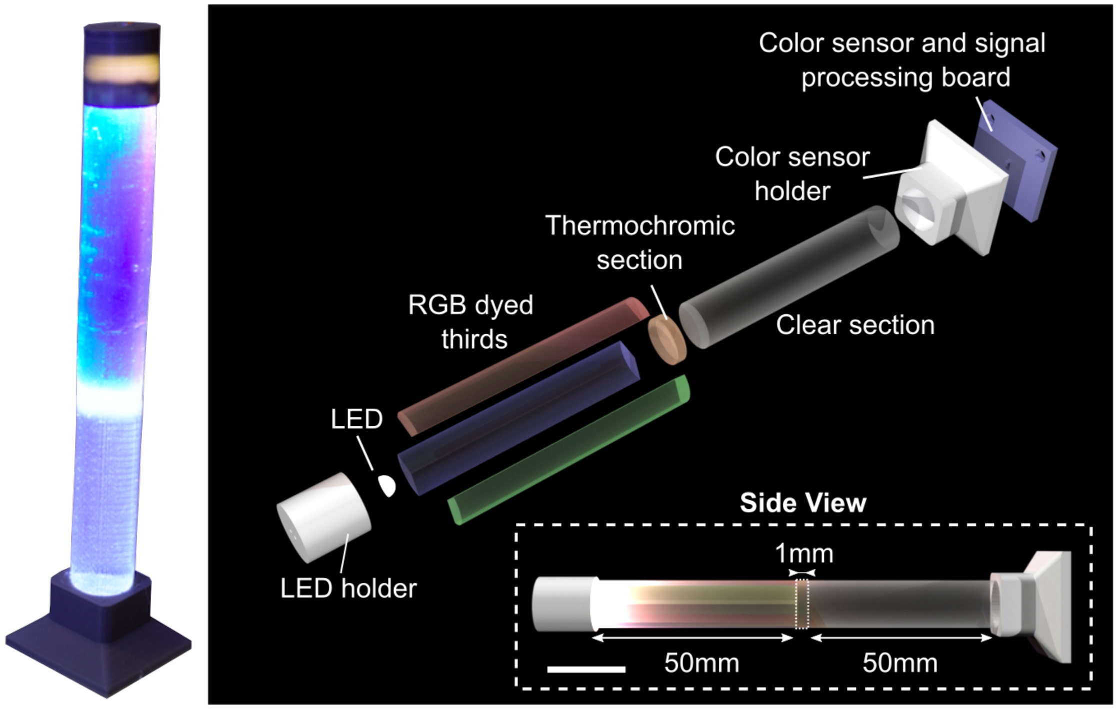

Supplementary Figure 1: **Specific ChromoSense embodiment details** Left: image of sensor in ambient lighting. Right: Exploded view and dimensions of specific sensor embodiment used for characterization. Scale bar on inset: 20 mm.

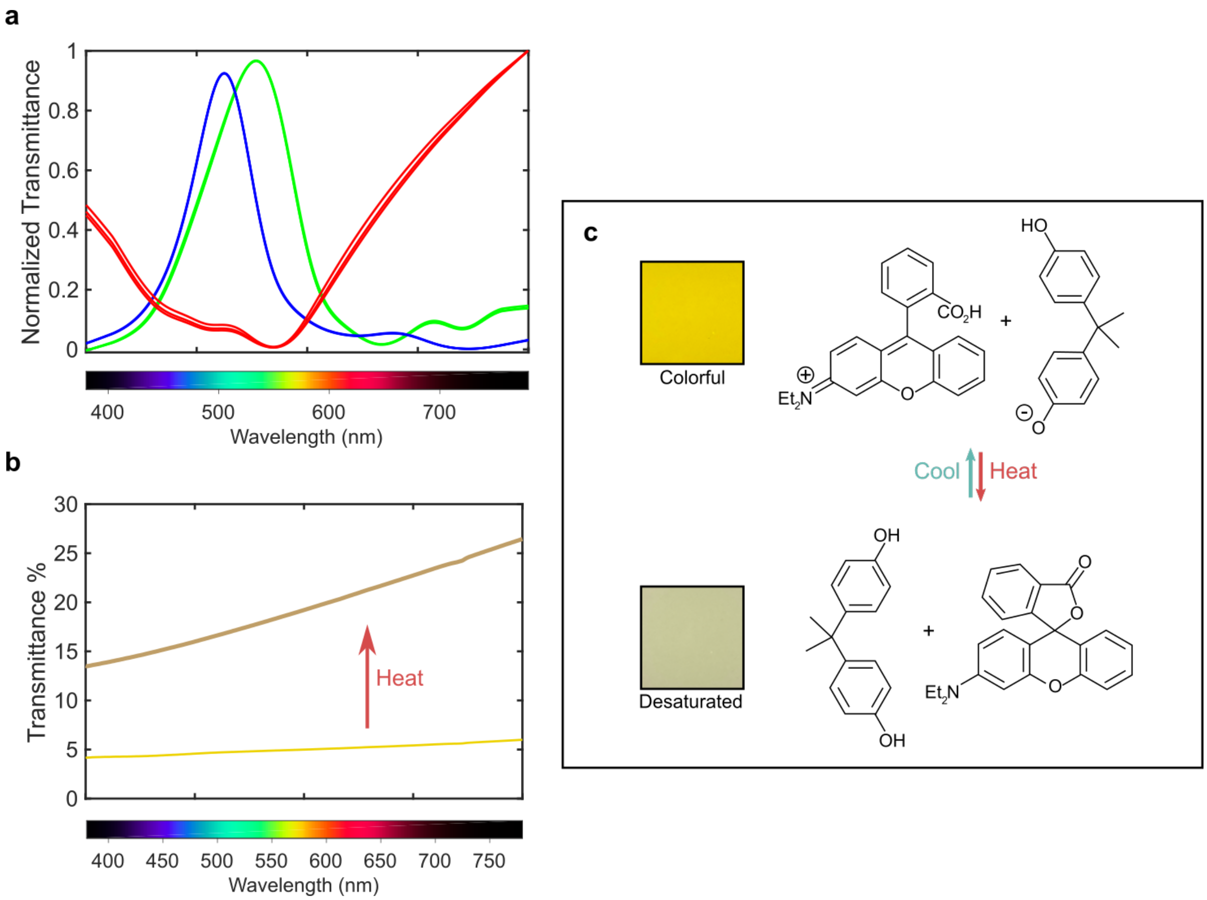

Supplementary Figure 2: **Characterization of dyes a.** UV-vis transmittance results for dyes used in sensor for demos. The color of the line corresponds to the color of the dye. **b.** UV-vis transmittance results for the thermochromic yellow dye. **c.** Left: optical images of 22°C and specimen heated above 31°C. Right: Chemistry of typical heat-induced color change exhibited by fluoran-based thermochromic compounds, adapted from [53].

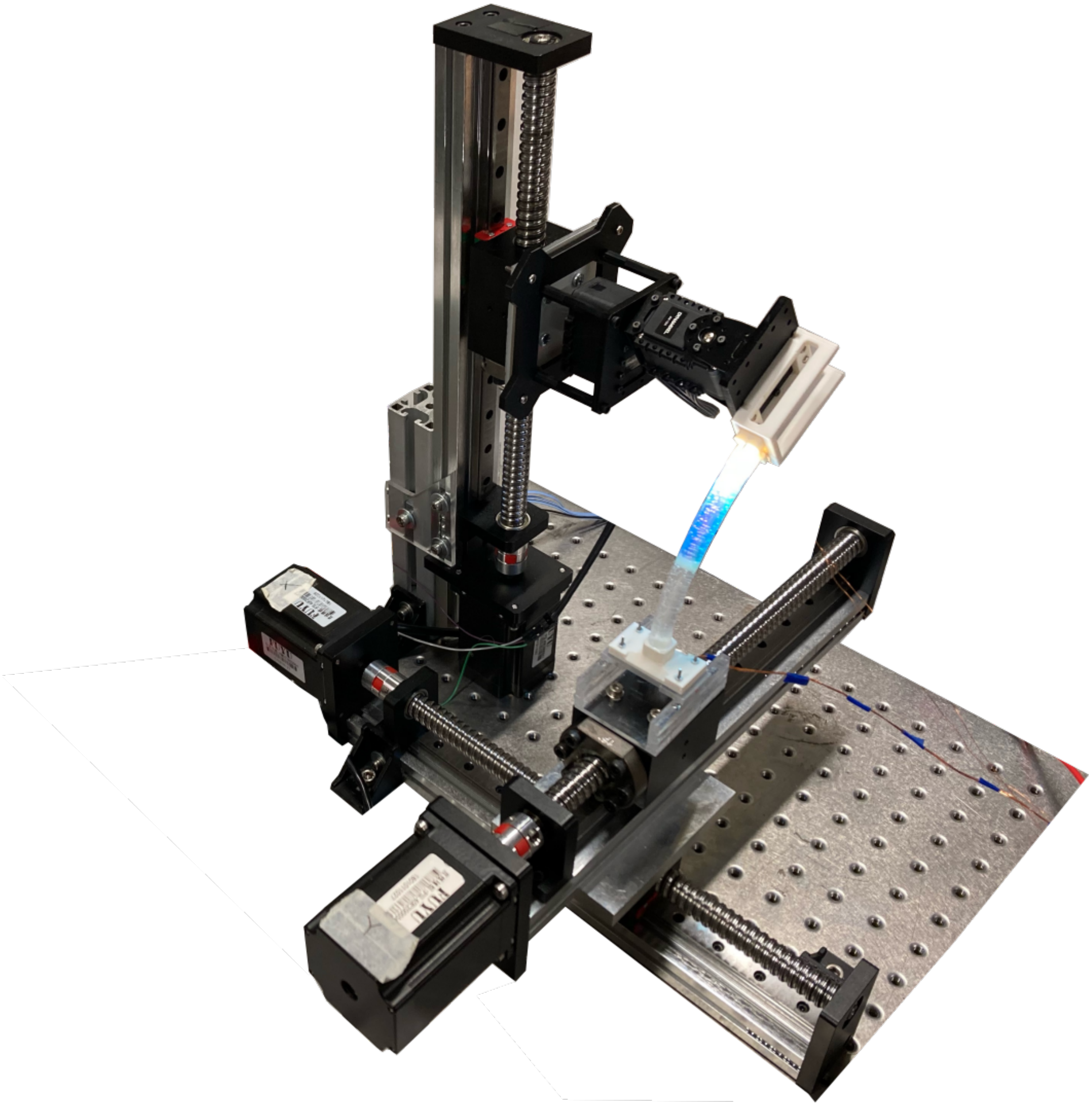

Supplementary Figure 3: **Multi-DoF deformation characterization setup** Setup used for subjecting sensor to a variety of deformation modes includes X, Y, Z, and roll and pitch degrees of freedom.

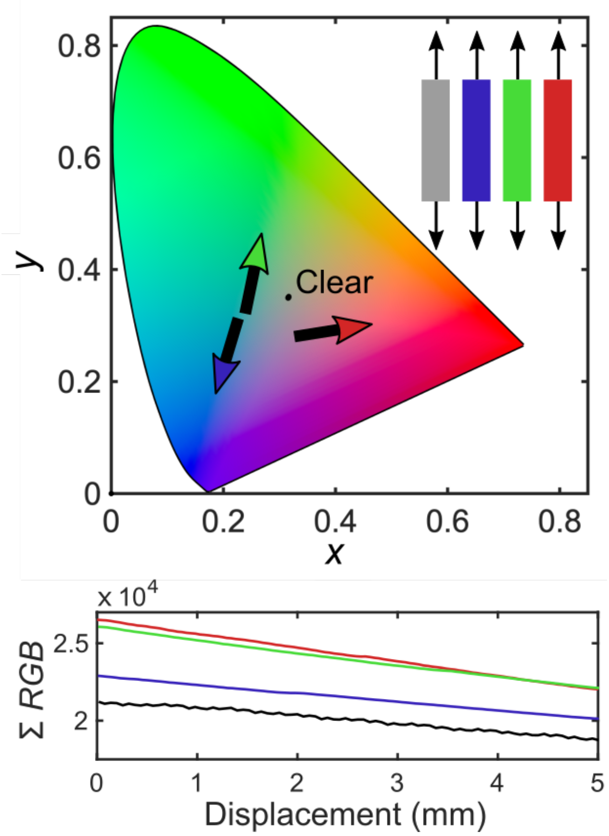

Supplementary Figure 4: **Beer's law validation tests** Tests conducted with a beam of a single dyed color, red, green, blue, or clear. Top: Vectors on CIE plot as specimens are pulled from 0 to 5 mm. Bottom: intensity of signal response as function of displacement.

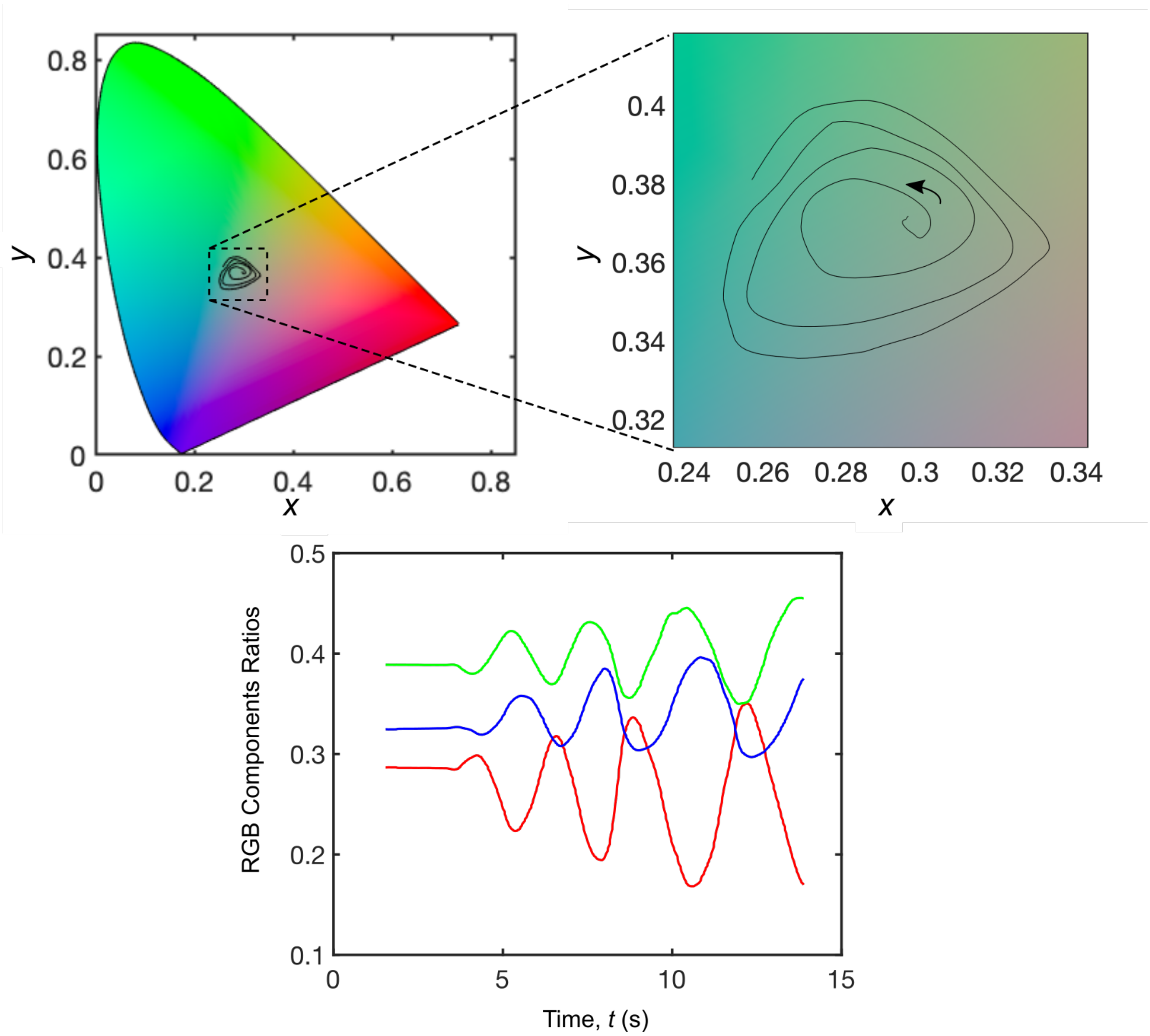

Supplementary Figure 5: **Continuous resolution and 1:1 mapping in bending** Top: Moving the sensor by hand in a spiral motion, the CIE diagram plots an analogous contour, illustrating 1) the continuous resolution in bending direction and magnitude of bending, and 2) the uniqueness of a bent configuration and RGB triplet values. Bottom: RGB component ratios are plotted as a function of time during the spiral motion.

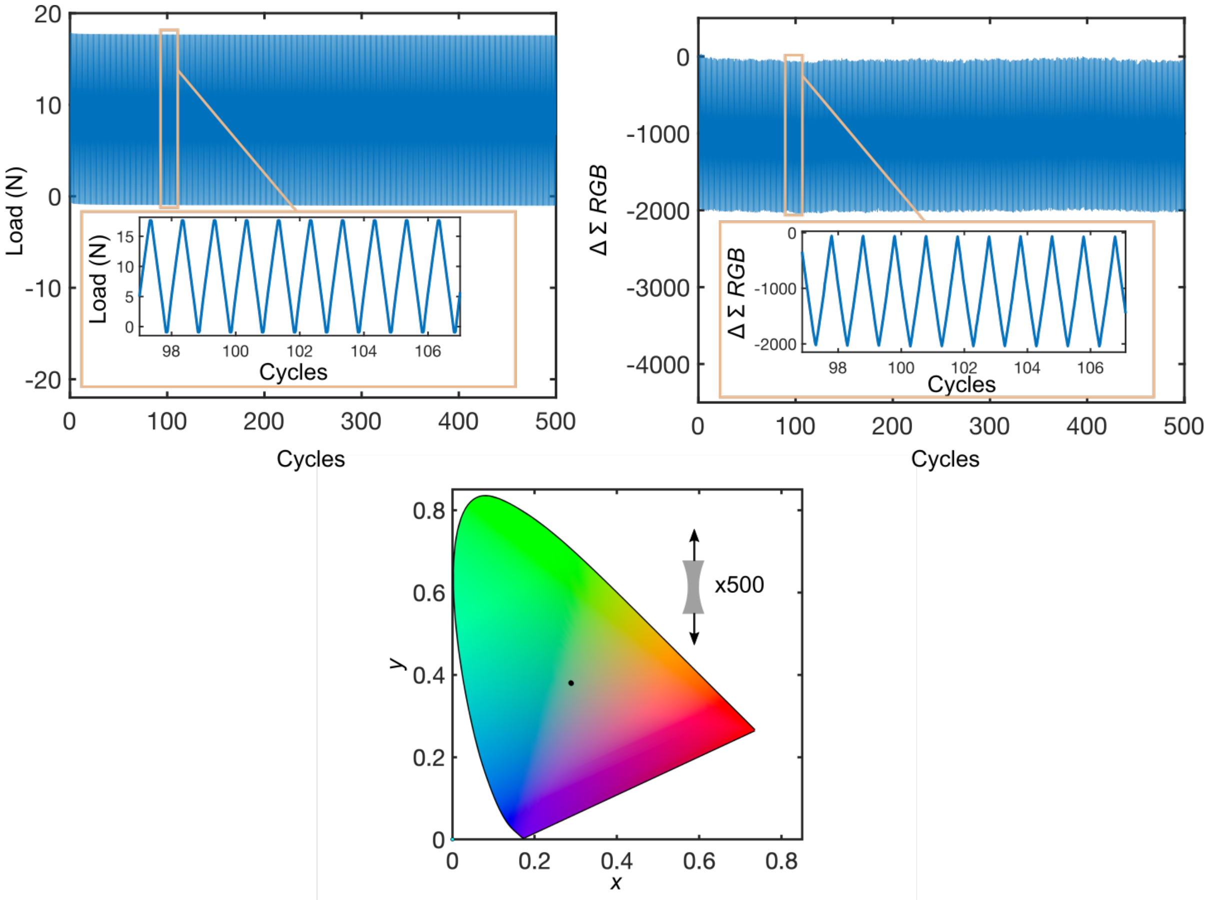

Supplementary Figure 6: **Cyclic tension test** Top: Force and intensity change as a function of cycle. Peak force corresponds to minimum intensity, as can be seen from the insets. Max strain was 10%. Bottom: chromaticity is plotted for the entire test, and shows minimal change.

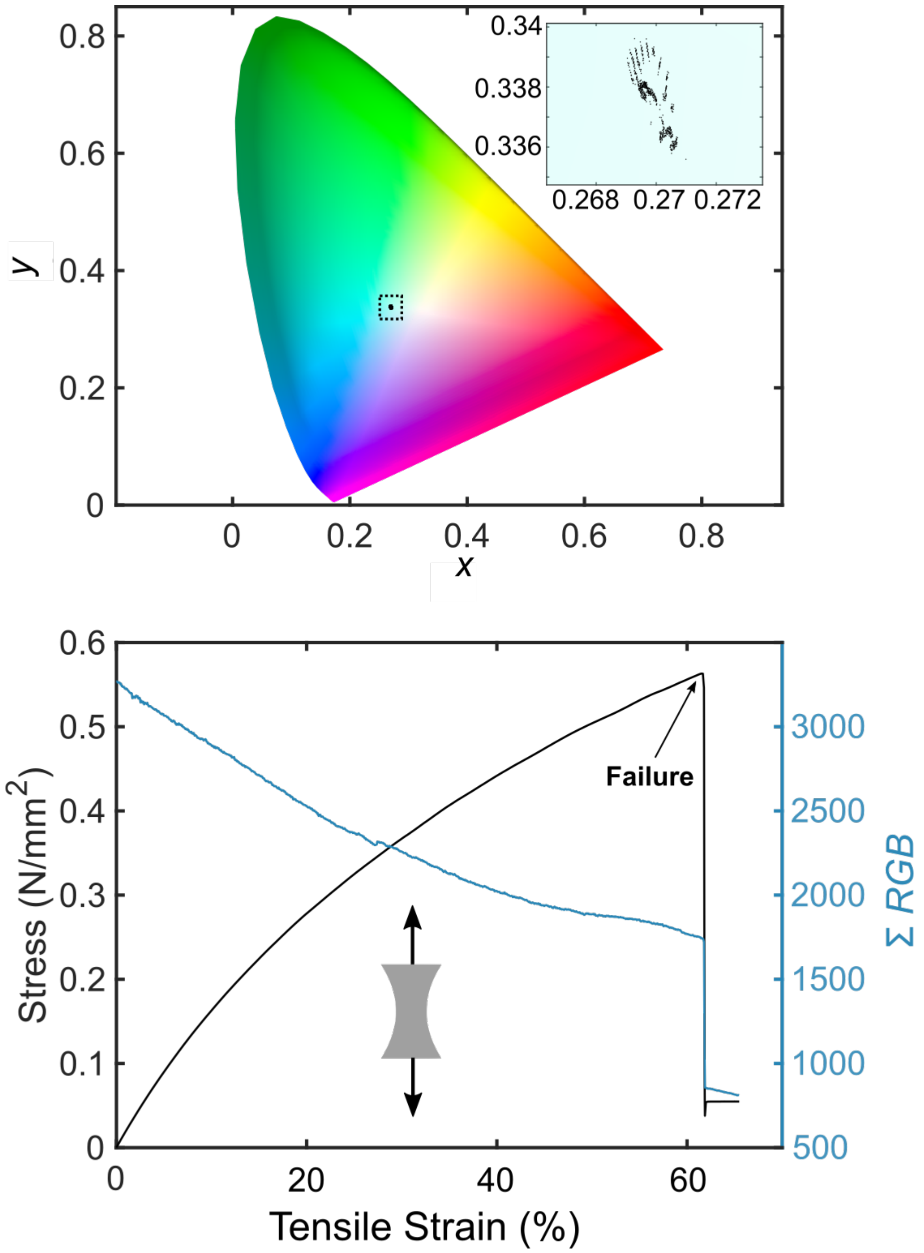

Supplementary Figure 7: **Pull-to-failure test of sensor** Top row shows chromaticity plot, and the bottom displays intensity and stress on different y axes as a function of tensile strain. Note that mechanical failure of the sensor coincides with a rapid decrease in intensity as the waveguide effect of the sensor is destroyed.

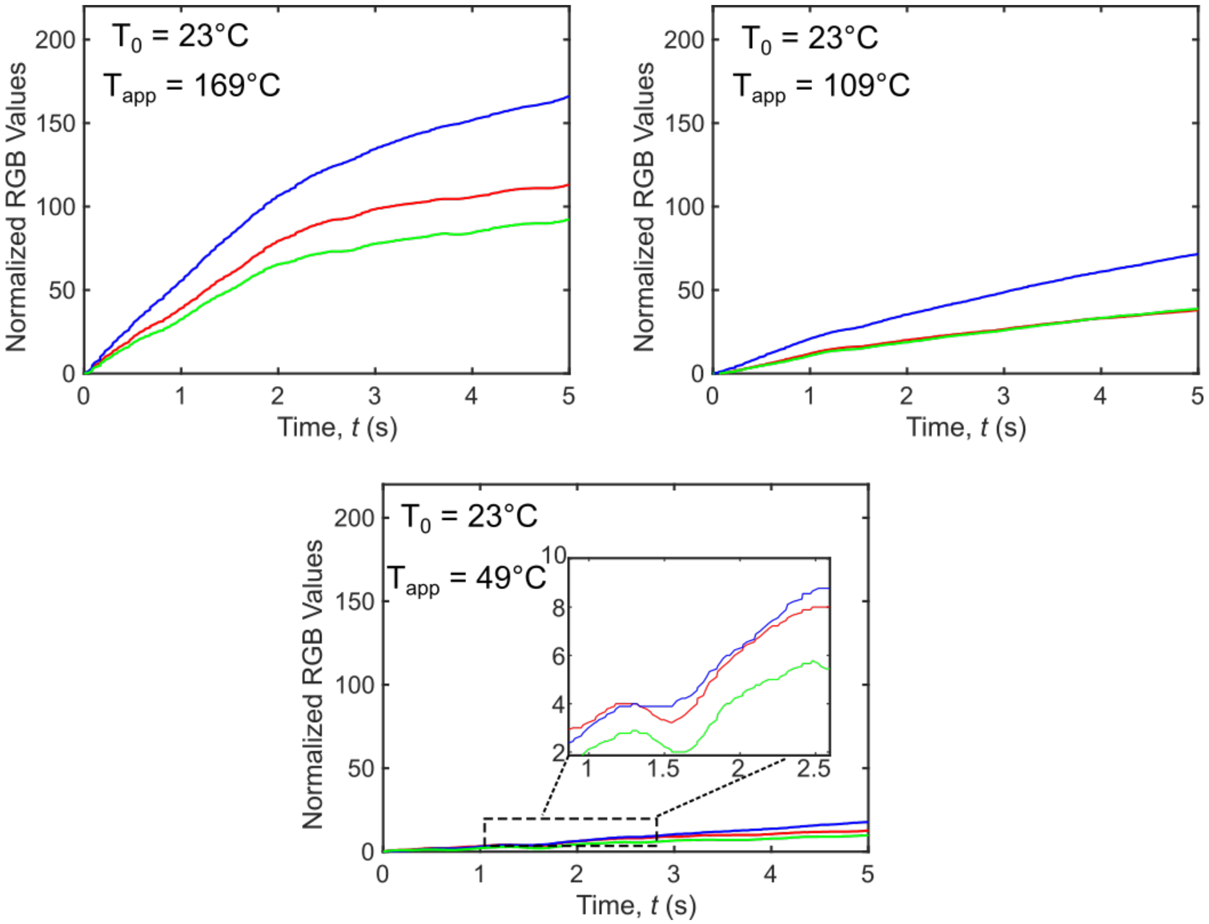

Supplementary Figure 8: **Effect of temperature on rate of color shift** Red, green, and blue raw component values (normalized to start at 0) are plotted as a function of time subjected to different temperatures for 5 sec. All tests started with the sensors at T_0 =23°C.

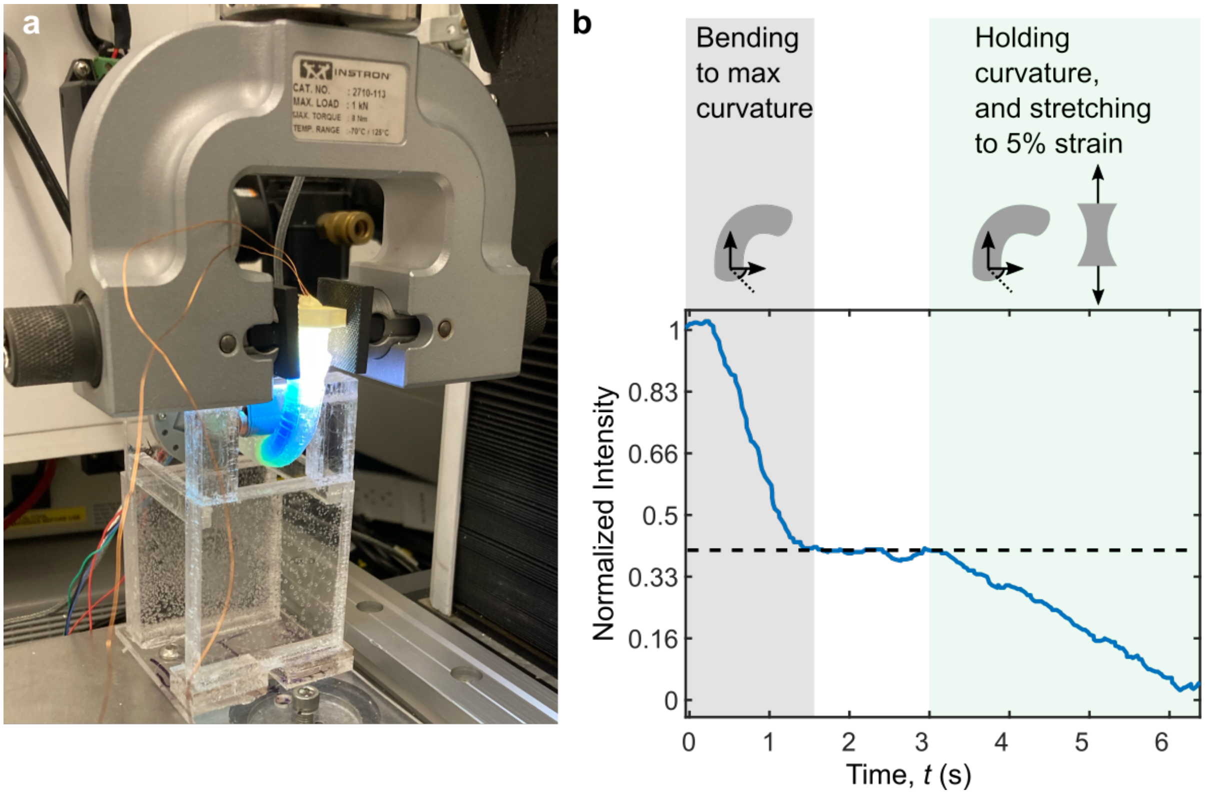

Supplementary Figure 9: **Combined Stretch and Bending a.** Custom fixture for combined stretch and bending. The sensor is bent at the maximum saturation curvature and then pulled from the Instron grips. **b.** Example intensity value plot ($\sum RGB$ normalized between 0 and 1) that facilitates decoupling stretch from bending when the deformations are applied sequentially. The average intensity value when purely bent decreases to a minimum value corresponding to the maximum curvature. Any further decreases in intensity result from further stretch, which can be decoupled from bending because stretch does not markedly change chromaticity. Crucially, this behavior also allows us to decouple intermediate deformations or any ordering of sequentially applied stretch and bending from the output signal: bending will always be accompanied by intensity and chromaticity changes together, while stretching will only have non-negligible intensity changes.
