# Supplementary material for: Multi-modal deformation and temperature sensing for context-sensitive machines
Source: Nat Commun. 2023 Nov 18;14:7499. doi: 10.1038/s41467-023-42655-y (PMC10657382; doi:10.1038/s41467-023-42655-y)
Supplement: Supplementary file 3 — Description of Additional Supplementary Files Document [file 41467_2023_42655_MOESM3_ESM.pdf]

Description of Additional Supplementary Files

Supplementary Movie 1: This video shows the sensor's color output change upon bending in different directions.

Supplementary Movie 2: This video shows the application of a sensorized soft exosuit.

Supplementary Movie 3: This video shows how one sensor can be used to create a digital twin of an origami interface.

Supplementary Movie 4: This video shows how the sensor enables an autonomous variable stiffness manipulator that processes both temperature and deformation stimuli.
